# Myocardial fibrosis imaging based on T1-mapping and extracellular volume fraction (ECV) measurement in muscular dystrophy patients: proof of additional diagnostic value compared to conventional late gadolinium enhancement (LGE) imaging

**DOI:** 10.1186/1532-429X-16-S1-P296

**Published:** 2014-01-16

**Authors:** Anca Florian, Anna Ludwig, Sabine Rösch, Handan Yildiz, Udo Sechtem, Ali Yilmaz

**Affiliations:** 1 Cardiology and Angiology, University Hospital Münster, Münster, Germany; 2 Cardiology, Robert-Bosch-Krankenhaus, Stuttgart, Germany

## Background

Cardiac involvement with progressive myocardial fibrosis leading to dilated cardiomyopathy is a major cause of death in muscular dystrophy patients. Extracellular volume fraction (ECV) measurement based on T1-mapping pre- and post-contrast promises the detection of early "diffuse" myocardial fibrosis that cannot be depicted by conventional contrast-imaging based on late gadolinium enhancement (LGE). With this study, we evaluated the presence of diffuse myocardial fibrosis in regions of "normal" (LGE-negative) and "diseased" (LGE-positive) appearing myocardium as well as its relation to the extent of left ventricular (LV) dysfunction and the occurrence of arrhythmias in Becker muscular dystrophy (BMD) patients.

## Methods

Twenty-seven BMD patients (35 ± 12 yrs) and 28 matched healthy CONTROLS (33 ± 8 yrs) underwent cardiovascular magnetic resonance (CMR) studies including ECV measurement and LGE-imaging. Ambulatory monitoring of arrhythmic events was performed by means of an external event loop recorder.

## Results

Twenty BMD patients (74%) demonstrated cardiac involvement as detected by typical inferolateral presence of LGE. Twelve patients (44%) had an impaired LV ejection fraction - all being LGE-positive. Global myocardial ECV was significantly higher in the BMD group (29 ± 6%) compared to the CONTROL group (25 ± 3%, p = 0.005). Patients with cardiac involvement demonstrated higher global ECV (31 ± 6%) as well as significantly increased regional ECV not only in LGE-positive segments (34 ± 6%), but also in LGE-negative segments (28 ± 6%) compared to BMD patients without cardiac involvement and to CONTROLS, respectively (24 ± 3% and 25 ± 3%, p = 0.01). Global ECV in patients with cardiac involvement substantially correlated to LV ejection fraction (r = -0.629, p = 0.003) and to the number of LGE-positive segments (r = 0.783, p < 0.001). On univariable analysis, global ECV - but not the categorical presence of LGE per se - was significantly associated with arrhythmic events (OR 1.97, CI 32.22-1.21, p = 0.032).

## Conclusions

ECV measurement by CMR is a useful tool in assessing the total extent of myocardial fibrosis as well as in depicting subtle diffuse fibrosis in areas of normal appearing myocardium on LGE-images. Thus, myocardial ECV is a potential additional quantitative tool for accurate detection of cardiac involvement and risk stratification in muscular dystrophy patients.

## Funding

This work was financially supported by a grant from the German Society of Cardiology (DGK; grant-ID DGK12/yilmaz to A.Y.) and by the Robert-Bosch-Foundation (grant-ID KKF-11-14 to A.Y.).

